# Medico-Chirurgical Transactions, Vol. XVIII

**Published:** 1835-04-01

**Authors:** 


### Medico-Chirurgical Transactions. Vol. XVIII.

### I. On Irritation of the Spinal Cord and its Nerves, in connexion with Disease in the Kidneys. By Edward Stanley, F.R.S., &c.

We owe an apology to the distinguished author of this paper, for neglecting to notice it at an earlier period than this. If, indeed we were disposed to review books and papers according to the new and fashionable recipe adopted by many critics—namely, by appending to each book a few lines of tart censure or honied praise, we could spread a catalogue of critiques each quarter before our readers, much more extensive than the catalogue of ships, and chiefs, and armies sent to the Trojan war by the Greeks of old. But this is not our custom. We present our subscribers with solid dishes—not soufflets, soup-maigres, or eau-sucrès, that fill the intellectual stomach with flatus, or tickle the palate without satisfying the appetite.

In this paper, Mr. Stanley relates cases of disease of the kidneys, co-existing with spinal tenderness and paraplegiac affections—which cases were treated as diseases of the fibro-cartilages and bodies of the vertebræ themselves.

The first case that attracted his attention was that of a man in Bartholomew's, admitted for paraplegia and retention of urine. Sensation and motion were both lost in the lower limbs. On examination, there was tenderness of the spine at the third lumbar vertebra, and this seemed to indicate the existence of disease, and, consequently, issues were inserted. Considerable amendment followed, with a certain degree of restoration, both of sensation and motion. The retention of urine, however, changed into incontinence. Here the improvement ceased—the general health failed—and he died.

On dissection, no disease could be discovered in the containing or contained parts of the vertebral column—all being perfectly sound, as were the brain and its membranes. In one kidney were numerous small abscesses—the other was gorged with blood and softened in texture. The mucous lining of the ureter and bladder was very vascular, and the muscular coat of the latter was thickened.

The next case occurred in 1821, in a man aged 35, admitted on account of partial loss of power, both in the upper and lower extremities, of a month's standing, and supposed to originate in disease of the cervical vertebræ. He suffered from irritation in the bladder, and occasional inability to expel the urine, which contained a puriform fluid. His health gradually declined, and he died six weeks after admission into Bartholomew's. On examination, both kidneys were found gorged with blood. In the substance of one, there were small purulent depôts. The muscular coat of the bladder was thickened, and its lining membrane very vascular. No morbid appearance in the brain or spinal cord—nor in the vertebræ.

The third case was communicated to Mr. Stanley by Mr. Hunt, of Dartmouth. A gentleman, aged 28, fell upon his back and bruised it, but soon recovered. Some weeks afterwards he was chilled, after being violently heated. On the following night, he was seized with a severe pain in the loins, partially relieved by bleeding. Some pain continued, and he began to lose sensation and motion in the lower limbs. The pain in the back was increased by pressure, but was not influenced by the application of a hot sponge. He gradually lost all motion and feeling in his limbs, together with the power of retaining his urine, and in this condition he died.

"On examination of the body, one kidney was found to consist of a mere sac distended with pus, and in the other kidney, were several small abscesses. There was no disease in the spine, or in any other part of the body." 263.

The fourth case was furnished by the late Mr. Horwood, of Gosport.

"The patient having suffered a fall upon his back which was followed by paralysis of the lower limbs, his complaint was, in consequence, considered to be disease of the spine, and the appropriate treatment was adopted and continued to the time of his death.

The appearances on dissection were, abscesses in both kidneys, thickening of the coats of the bladder, with enlargement of the prostate gland. The thoracic and abdominal viscera were healthy. The brain and spinal cord were healthy excepting an unusual vascularity in the membranes of the latter below the first lumbar vertebra. There had not been the slightest suspicion of disease in the kidney, the pain in the back having been wholly referred to the disease which was supposed to exist in the spine.

Here were four cases considered to be diseases of the spine, and treated accordingly, in which the vertebræ, their fibro-cartilages and ligaments were found healthy, and the only disease ascertained to exist was seated in the kidney, with which, therefore, we are to presume the irritation of the spinal cord was connected. To the question whether the irritation of the spinal cord might have been an independent affection with which the disease in the kidney was casually coincident, the proper reply will be presently considered." 264.

Mr. Burnett, house-surgeon of Bartholomew's drew up the particulars of the fifth case. A man, aged 22 years, was admitted for retention of urine, the consequence of a severe gonorrhœa, stopped by injections. The bladder then lost its expulsive power—the sphincter ani became paralytic—partial paralysis of the lower extremities. Complained of severe pain in the back, about the fifth lumbar vertebra. His countenance was flushed and anxious—tongue furred—pulse 120—abdomen tense and tender, especially in the hypogastric region—and he distinctly traced the course of his pain from the bladder upwards to the left kidney, then across his loins to the right kidney. He soon lost power and feeling in the lower extremities. The urine dribbled away, and the fæces passed involuntarily. The urine drawn off from the bladder was dark-coloured and offensive, containing mucus. Various remedies were used, with little or no effect. He died about a fortnight after entering the hospital.

"On examination of the body, the kidneys were found larger than natural, and of a very soft consistence. Sections of them discovered, with the turgescence of the bloodvessels, numerous minute depositions of pus throughout both the cortical and tubular parts. The infundibula and pelves were filled with pus mixed with a thick, ropy mucus. The mucous membrane lining the bladder was very vascular, and in part covered by coagulable lymph. There was no morbid appearance discoverable in any part of the brain or spinal cord." 266.

In the above, as in the four other cases, there was nephritis, with paralysis of the lower extremities; but the symptoms were less equivocal, and the disease of kidney could scarcely be overlooked.

In the course of May, 1833, the following case occurred in St. Bartholomew's under the care of Mr. Earle. A man, aged 30, was admitted for gonorrhœa and phymosis. The discharge continued, though the inflammation had subsided. In this state, and without any apparent cause, he was seized with paraplegia, from the umbilicus downwards. The functions of the brain were undisturbed. He stated that he had been suffering for a day or two from pain in the loins. Cupping and purgation gave no relief. The urine flowed involuntarily. A catheter was introduced, and brought away three pints of urine. In sixteen hours from the commencement of the attack, he suddenly fell back in his bed and died.

"With the recollection of the case last related, I ventured to predict that in this instance, we should find inflammation in the kidneys. The spinal cord was first carefully examined. There was found some turgescence of the vessels, both in the membranes and substance of its lumbar portion, and a few drachms of transparent fluid in the theca, but neither the turgescence of vessels, nor the effusion of fluid were sufficient to explain the paraplegia by pressure on the cord. The liver was enlarged and indurated. The other abdominal viscera, with the exception of the kidneys, were sound, and with no unusual turgescence of the vessels. Both kidneys were of so dark a colour as to be almost black; they were remarkably flaccid, and on sections being made of them, were found to be in every part gorged with blood. The mucous lining of the infundibula and pelves was dark-coloured from the turgescence of the vessels. The coats of the ureters and the mucous lining of the bladder were also very much more loaded with vessels than is usual. In the bladder was about a pint of urine. Some fluid was found between the membranes of the brain and in its ventricles.

When the circumstances of the foregoing case are viewed with the other cases which preceded it, it seems reasonable to believe there was a connexion between the occurrence of the paraplegia, and the inflammatory state of the kidneys evidenced by the great determination of blood to their substance. Had life endured for a longer period, it is probable there would have been suppuration in the kidneys. From the period of the occurrence of the paraplegia there was an inordinate secretion of urine, which is worthy of remark in connexion with the great determination of blood to the kidneys." 268.

Dr. Burrows furnished our author with some particulars of a case that occurred in the same hospital under the care of Dr. Latham. A man, aged 35, was admitted with incontinence of urine and severe pain in the back, which had existed for two years. The pain extended from the first dorsal to the last lumbar vertebra, being most severe at the sixth dorsal vertebra, and increased by pressure. There was pain over the front of the chest, and dyspnœa. Urine and fæces passed involuntarily—pulse 80. Abstraction of blood proved of no use, except to the dyspnœa. He gradually sunk. A considerable quantity of serum was found beneath the arachnoid in the head, as also in the theca vertebralis; and the pia mater covering the lumbar portion of the spinal cord was very vascular. In other respects the cord was healthy, as were the thoracic and abdominal viscera, with the exception of the kidneys. The pelvis and infundibula of the right one were dilated and distended by a thin puriform fluid, and, in some situations, the substance of the kidney had been absorbed, so that the infundibula extended almost to its outer surface. The rest of the substance of the kidney presented a mottled appearance from alternation of white and red spots. The other kidney shewed a similar appearance. The bladder contained a pint of thin, puriform fluid, and its mucous lining was thickened.

"In subsequent communications with which I have been favoured from Mr. Henry Hunt of Dartmouth, he states that he had attended four cases of disease in the kidney existing in connexion with symptoms of affection of the spine, and that 'the peculiarities of these cases appear to him to be, first, that the symptoms simulate those of the incipient stage of inflammation of the vertebræ, thus there are the numbness, cramps, and inability of commanding the legs. 2dly, that there is a peculiar feeling of tight wires or cords in different directions through the limbs. 3dly, that with the first stage of the inflammatory attack in the kidney, the urine is not altered in quantity or quality, and consequently it was pronounced that no disease in the kidney existed until it was indicated by the mixture of pus with the urine.' Mr. Hunt further alludes to the occurrence of cases of disordered uterus combined with loss of power in the lower limbs in such a degree, that the patients were wholly confined to their beds, adding that, by the subsequent and perfect recovery of some of these patients, it was clearly proved there had been no change of structure in the parts to which the symptoms referred as the source of irritation." 270.

It can hardly be supposed that the disease in the kidney and irritation of the spinal cord, in all these cases, were purely coincidences, and unconnected as cause and effect. In the foregoing cases it seems probable that the disease commenced in the kidney, and extended to the medulla spinalis and its nerves afterwards. The reverse of this is a catenation every day observed—namely, irritation of the spinal cord being communicated to the kidneys and other parts of the urinary apparatus.

"The lumbar ganglia of the sympathetic communicate freely with the spinal nerves, and from these ganglia, nerves issue to the renal plexus. From this arrangement of nerves, an explanation may be derived of the influence of the spinal cord upon the functions of the kidney; still more closely can we trace the influence of the spinal cord upon the bladder, rectum, and uterus, through the nerves which these organs receive directly from the sacral plexus of spinal nerves." 272.

Dr. Prout informs us, that in a large proportion of cases, the deposition of the earthy phosphates from the urine, has been traced to injuries of the spine, from concussion or falls. It is well known that the more serious injuries, as fracture or dislocation of the spine, frequently prove fatal by inducing inflammation of the mucous lining of the bladder, or rather by producing the cause of that inflammation—ammoniacal urine. Our author thinks that the irritable uterus described by Dr. Gooch is, in many instances, dependent on irritation of the spinal nerves; for we see great benefit, in such cases, from issues in the loins. The following interesting case of irritable bladder we shall extract.

"Charlotte Sepping, æt. 25, was admitted into St. Bartholomew's Hospital with the following symptoms. Pain and tenderness in the side just above the crista of the ilium. Urine very scanty and high coloured, and voided with great distress. She stated that she had been ill about a month, and that at the beginning of her illness, after straining to empty the bladder, she felt a sensation of something giving way in her side, and immediately passed a teacupfull of blood. She had occasionally passed gravel. She suffered most severely from pain in the bladder, and required the constant introduction of the catheter. From the suspicion there might be a stone in the bladder, she was sounded by Mr. Abernethy, who pronounced there was no stone, but that the bladder was very irritable, and without the power of contracting. Every form of medicine that was likely to relieve the irritability of the bladder was tried, but ineffectually; her sufferings continued, the pain in the bladder was excessive, there was also severe pain in the lower part of the back, and the urine continued to be mixed with clots of blood. On examining the spine, acute tenderness was discovered in the spinous processes of the lower lumbar vertebræ, and an issue was accordingly made in this situation. As soon as the discharge from the issue commenced, her sufferings began to subside, the bladder became gradually tranquil, and in about a month, she left the hospital perfectly well, and it was afterwards ascertained that she had no return of the complaint." 277.

Mr. Stanley enters into some physiological speculations respecting the mode in which the irritation is propagated from the kidney or bladder to spinal nerves, and through them to parts where both sense and motion are lost or impaired. Some experiments of Mr. Mayo are referred to, and then he remarks.

"In the instances which have been adduced, irritation commencing in the nerves of an internal organ, the kidney or bladder, has been transmitted through the spinal cord to the motive and sentient nerves of the limbs; but the same phenomena may occur in an opposite order, as in the case of a compound fracture or other severe injury of the lower extremity followed by retention of urine, from irritation arising in the anterior crural and ischiatic nerves, and communicated through the lumbar and sacral plexuses of spinal nerves to the nerves of the bladder. Extending these views to cases of neuralgia, where there is no visible derangement of structure, or other local cause of excitement, it will always be difficult to determine whether the source of irritation be in the affected nerves, or in the central portion of the nervous system whence they are derived. In one case of neuralgia affecting the nerves of the thigh, after ten years of severe suffering, the patient died, and upon dissection, with a perfectly healthy condition of every other organ, the lumbar portion of the spinal cord on its posterior surface, was found covered with numerous large, but thin plates of cartilaginous substance, deposited in the arachnoid membrane. Here it must be, I conceive, difficult to state whether the morbid condition of the spinal cord bore the relation of cause or effect to the painful affection of the nerves of the thigh." 279.

This paper, "which is simply intended to illustrate, by a variety of facts, the reciprocal connexion between the kidneys and the spinal cord," is creditable to Mr. Stanley, and cannot fail to attract the attention of practitioners to a very important train of investigation.

II. ON MALIGNANT TUMORS CONNECTED WITH THE HEART AND LUNGS. By JOHN SIMS, M.D. Physician to St. Mary-le-bone Infirmary.

Four cases are detailed by Dr. Sims, in whose talent for observation, and accuracy of narrative, we place the greatest dependence. The growth of morbid or malignant tumors in various tissues of the body, has occupied much attention of late years; but few cases are recorded where the heart was involved in the diseased process. The first two cases in this paper belong to that class of tumors denominated fungoid, medullary, soft cancer, &c. and present some interesting circumstances.

*Case* 1. This was a complicated and distressing case. A female, aged 23, had enjoyed good health till the commencement of the present disease. Recently she complained of dyspnœa, cough, pain in the chest, and symptoms supposed to indicate inflammatory action, which did not, however, give way to the usual remedies. She mentioned to Dr. S. a swelling in the lower part of the abdomen, and, on examination, several distinct and large tumors could be felt rising out of the pelvis. "Above the clavicle, and along the blood-vessels of the right side of the neck, there was a number of enlarged lymphatic glands." Her disease made a rapid progress—fluid collected in the cavity of the peritoneum—the tumors rose higher and enlarged—the limbs became œdematous—the dyspnœa augmented, as did the cough, but without expectoration.

"A remarkable symptom now occurred: the sounds of the ventricles were perceived in their usual situation, but the impulse of one or both ventricles was equally distinct over a considerable part of the right side of the thorax anteriorly. Her right arm became painful and enormously swoln, presenting all the signs of phlegmasia dolens from inflamed veins." 283.

The sacrum sloughed, and death terminated her sufferings.

*Dissection.* The head was not examined.

"*Thorax.*—On opening the thorax, a tumour of very considerable size was found imbedded in the right lung, it was closely attached to the great vessels at the base of the heart; it was moveable within the thorax.

On making sections through it, some portions appeared firm and fibrous, and others softer and brain-like. Its colour was of a dirty white, intermixed with streaks of a lead colour, apparently in the direction of its few blood-vessels. It closely involved the bronchi and blood-vessels at the root of the right lung, and was firmly attached to the pericardium and vessels immediately issuing from the heart. Circumstances did not allow me to remove the tumour.

Nothing remarkable was observed in the left lung or the texture of the heart.

On dissecting out the right subclavian vein, the preparation of which is now before the Society, it was found to be filled with successive layers of fibrine, the product of inflammation, and the valves at its junction with the jugular are seen distended with this deposit.

The cavity of the peritoneum contained several pints of fluid. The viscera had a deep leaden hue, and there was a remarkably strong exhalation of carburetted hydrogen gas. There were several very large tumours attached to the uterus and its appendages, some of them the size of large oranges: they were soft, and their texture was exactly analogous to the tumour found in the lung." 285.

We beg to draw the attention of our readers to the following passage.

"I need not relate the various means that were used to mitigate the sufferings of this young person. I may however remark, that needle punctures, made along the inner side of the upper extremity, *materially relieved the pain and nearly reduced the swelling*: for, as will be shewn, this was a true specimen of phlegmasia dolens, and I am not aware that this remedy is had recourse to in the analogous affection of puerperal women." 284.

Now, with all due deference to Dr. Sims, we beg to enter our caveat against the statement that this was a "true specimen of phlegmasia dolens." It was a case, undoubtedly, of phlebitis and its consequence, œdema—but very different from the comparatively rare disease described under the name of phlegmasia dolens. In the *latter* the swelling is tense and elastic. The part pressed by the finger quickly rises to its accustomed level—and the fluid will *not* drain off by any number of punctures made by the needle. All the disputes that have arisen respecting the pathology of the disease, have been occasioned by confounding the more common malady—phlebitis—with the more rare one—phlegmasia dolens. For one case of the *latter* we have witnessed more than ten of the *former.* They may easily be distinguished from each other. The swelling in one case is elastic, in the other œdematous. The phlebitis is very dangerous and often fatal—phlegmasia dolens is hardly ever fatal. No wonder then that the pathology of the one should be unknown, and that of the other so well ascertained by multiplied dissections.

In the above case, the impulse communicated from the heart along the tumor was curious, and might have readily led to false diagnosis, had not auscultation lent its assistance.

*Case* 2. This was a malignant tumor affecting the right lung, and penetrating the left auricle of the heart. John Imber, aged 43, a baker, applied to Dr. S. twelve months ago, for an attack of hæmoptysis, attended with symptoms indicating a loaded state of the thoracic vessels. He had several subsequent attacks, which were relieved by general and local bleeding, digitalis, blisters, and the usual means. Dr. S. then lost sight of him for several months. He was admitted into the infirmary on the 30th Oct.; meanwhile, his symptoms had undergone a material change for the worse. He had had several attacks of hæmoptysis—the dyspnœa was augmented—cough troublesome, with mucous expectoration. There was dulness on percussion over a considerable portion of the right side of the thorax anteriorly, and respiration was inaudible there. The jugulars were dilated to three times their size, and, with the subclavians, presented large tumors above the clavicles—face swollen—severe headache—pulse sharp. Bleeding, blisters, counter-irritation, &c. afforded some relief, but it was transient. He lingered, however, till the 28th December, when he died. The dissection is very minutely detailed, but we must pass over much of it.

" *Thorax.*—On raising the anterior parietes of the thorax, a portion of the tumour, several inches in circumference, came into view on the right side. The tumours of the right lung occupied about two-thirds of the capacity of the entire thorax. The diaphragm was lower than usual, the space for the left lung was encroached upon by the contents of the opposite side. The heart was situated several inches lower than usual, and pushed much beyond the mesial line.

The contents of the thorax, the liver, and diaphragm were now removed from the body.

*Left Lung.*—The left lung was free from adhesion, and the pleura of natural appearance; there was much black matter deposited, and some emphysema. This lung was considerably indurated in some parts, which, on being cut into, consisted of extensive red hepatization. On a careful examination of the lung, it was found to be quite free from the morbid growth contained in the other cavity of the thorax: the larger vessels and the bronchi were not engaged in the disease, although the tumours in the left auricle lay close to them.

*Right Lung.*—Pleura much thickened. This lung occupied a considerable space, for the augmented contents of the thorax had encroached upon the cavity of the abdomen. A great proportion of it was consolidated, apparently in consequence of old hepatization; in some parts the substance crumbled on the application of gentle pressure, this portion was of a dark or dusky red colour; there was pus in a few small cavities in the section. A small portion was comparatively healthy, and in degree fit for the purposes of respiration. The tumour was extensively attached to this lung, and portions had insinuated themselves between the larger vessels and the carnifications of the bronchi.

The *trachea* was so pressed upon by the tumour as to render the musculo-membranous part quite flat, and to expand the cartilages into a much wider arch. The bronchi, at the bifurcation, were much dilated; the right bronchus, with several of its subdivisions, passed directly through the tumour." 289.

The pericardium was much dilated at the base, by several large tumors developed within it. A tumor pressed upon the right auricle, so as to burst it inwards. The cavity was much dilated. The descending cava was much increased in length, and passed through the tumor. The cavity of the right ventricle was contracted. The right branch of the pulmonary artery passed through the tumor, and was much dilated. In the left auricle, the tumor had made much progress—the substance of the auricle being absorbed to a considerable extent. The left ventricle was small. The tumor occupied about one-third of the thoracic cavity, extending from the anterior to the posterior parietes, being formed of several lobes of various sizes—attached to the trachea, bronchi, heart, great vessels, and right lung. It had no capsule, but was merely covered by a thin membrane, except where it was attached to circumjacent viscera. Its consistence was various—some parts resembling soft cartilage—others scirrhus—some soft and pulpy, &c. and often containing a milky fluid like cream. The whole tumor very much resembled the oak-apple. The liver was much enlarged, and of a nutmeg appearance. There was no disease in any part of the alimentary canal.

"The immense tumour found in the thorax of Imber does not consist in an alteration of any material structure, but is a new formation or adventitious growth.

The organ or tissue in which it commenced, may admit of various explanation. My impression is that it began in the lung, in the vicinity of the great vessels, and subsequently extended itself in other directions. In the firmest part, where the bloodvessels going into it are most numerous, the tumour adheres intimately to the lung. In this part, either in the substance of the lung or in the filamentous tissue connecting it with the adjoining parts, the tumour most probably originated, and the more soft and pulpy portions connected with the heart and great vessels may have been subsequently, and more rapidly formed." 295.

*Case* 3. Sarah Fyfe, aged 58, admitted June 25th, 1831, complaining of pain in the abdomen, which is hard and tumid in the hypogastric region—urine scanty and high-coloured—surface of the body pale and bloodless—several small, hard, and moveable tumors in various parts of the body. Six weeks previous to admission, she had a profuse sanguineous discharge from the vagina, since which her health has rapidly declined. 27th. Examined *per vaginam*, and discovered round, indurated masses, about the os uteri. She lingered till the 4th of July, when she died.

*Dissection.* "*Head.*—A small quantity of fluid in the subarachnoid tissue and in the ventricles. Brain firm and remarkably pale: little or no blood appeared in the central or superficial vessels. A small indurated tumour in the falx of the dura mater. A tumour of a similar kind was situated in the right half of the pituitary gland, the size of a large pea. The carotid arteries in their canal were dilated and ossified. The posterior clynoid processes were thin and eroded, probably from the pressure of the arteries and the tumour of the pituitary gland.

*Thorax.*—Many tubercles were situated beneath the anterior part of the pleura costalis. Numerous similar tubercles were found on the pleura pulmonales, throughout the substance of the lungs. The lungs were edematous and studded with small miliary tubercles, and in the upper part of the right lung there were several cavities. A considerable quantity of fluid was found in the cavities of the pleuræ.

*Heart.*—Slight hypertrophy: the left ventricle was dilated and softened.

*Right Auricle.*—In the appendix to the right auricle there was a mass of adventitious deposit, the size of a small walnut.

*Abdomen and Pelvis.*—The liver and pancreas contained many tubercles of the character before mentioned: these with the other viscera of the abdomen, were in other respects natural.

*Uterus.*—The cervix was enlarged and indurated, and there was a small tumour projecting from the fundus into the cavity.

The filamentous tissue between the bladder and uterus was a good deal thickened and indurated, and of a similar appearance to that of the neck of the uterus.

*Bladder.*—The mucous membrane was generally vascular: about the anterior part of the neck large portions of the mucous membrane were detached, leaving a rough and vascular surface." 298.

*Case* 4. Charles Jones, aged 64, has been hemiplegiac of the left side for 12 months past. Was admitted July 23d, 1833. He had been subject to cough and other pectoral complaints for several years; but, owing to the urgency of other symptoms (not mentioned) no particular attention was paid to them. He died on the 6th of August following.

" *Dissection. Brain.*—An extraordinary morbid alteration was observed in the pons varolii; the particulars of the dissection of the brain, together with the symptoms referrible to it, I intend to relate on a future occasion.

*Thorax.*—In the upper portion of one of the lobes of the left lung was situated a tumour, the size of a small orange, it was imbedded in the centre of the lung, in the immediate vicinity of the bronchi and great vessel. Its surface was uneven with numerous rounded eminences. On making a section of the tumour, it proved to be of the fungoid or medullary character, and was composed of smaller lobules, in some parts presenting a high degree of vascularity. The lungs were edematous and loaded with blood. Traces of chronic pneumonia, in various stages, were observed, with copious infiltration of greenish fluid into the pulmonary tissue, in some places resembling gangrenous patches. There was extensive solid grey hepatization near the root of the left lung, where the tumour was situated.

Heart flabby, cavities and valves natural.

In the abdominal viscera no morbid change was noticed, except a large scrotal hernia, in which the intestine was confined by strong lengthened bands of membrane." 300.

We shall be anxious to learn the nature of the disease in the pons varolii, and the symptoms which attended it; but cannot expect to have our curiosity gratified till the appearance of the next volume of the Society's Transactions.

By the way, we have to congratulate the Society on two events—of unequal importance—first, the acquisition of a royal charter, by which they are enabled to make laws and regulations, the same as before, for their members. *Secondly,* the removal from Lincoln's-Inn Fields to the anomalous locality of Berners-street—a kind of " *no-man's-land,*" equidistant from the bustle of the city and the ennui of the court end. We do not think the situation chosen is the best that could have been found; but still it is better—

that is, more convenient, than its former locale. We hope the new title and the new house will greatly augment the number of the Society's FELLOWS—for certain it is, that some of its *members* have *fallen off* within the last two or three years, without an adequate growth of new ones. The *Royal* Medico-Chirurgical Society is a very useful one for the reading and publishing of long papers, that could neither be read nor discussed in the *plebeian* societies of the metropolis. The *latter*, however, will always have a decided advantage over the former in point of interest, and perhaps of utility. Cold water has generally been thrown on *discussion* in the aristocratic society, while it is the very life and soul of the popular associations. As all the good papers read at the Medico-Chirurgical Society are subsequently published, there is less anxiety to attend the rehearsal than otherwise would be manifested; while the fresh facts and collisions of opinion at the minor establishments keep up a constant curiosity, not to say excitement, in the minds of the listeners as well as of the debaters.

